# Activities of genes controlling sphingolipid metabolism in human fibroblasts treated with flavonoids

**DOI:** 10.1007/s11011-015-9705-x

**Published:** 2015-07-26

**Authors:** Marta Moskot, Joanna Jakóbkiewicz-Banecka, Elwira Smolińska, Bogdan Banecki, Grzegorz Węgrzyn, Magdalena Gabig-Cimińska

**Affiliations:** 1 Laboratory of Molecular Biology (affiliated with the University of Gdańsk), Institute of Biochemistry and Biophysics, Polish Academy of Sciences, Wita Stwosza 59, 80-308 Gdańsk, Poland; 2 Department of Molecular Biology, University of Gdańsk, Wita Stwosza 59, 80-308 Gdańsk, Poland; 3 Department of Molecular and Cellular Biology, Intercollegiate Faculty of Biotechnology UG-MUG, Kładki 24, 80-822 Gdańsk, Poland

**Keywords:** Gene expression regulation, Flavonoid, Lysosomal storage disease, Sphingolipid metabolism

## Abstract

Natural flavonoids such as genistein, kaempferol and daidzein were previously found to be able to reduce efficiency of glycosaminoglycan synthesis in cells of patients suffering from mucopolysaccharidoses, inherited metabolic diseases with often brain disease symptoms. This feature was employed to test these compounds as potential drugs for treatment other neuronopathic lysosomal storage disorders, in which errors in sphingolipid metabolism occur. In this report, on the basis of DNA microarray analyses and quantitative real time PCR experiments, we present evidence that these compounds modify expression of genes coding for enzymes required for metabolism of sphingolipids in human dermal fibroblasts (HDFa). Expression of several genes involved in sphingolipid synthesis was impaired by tested flavonoids. Therefore, it is tempting to speculate that they may be considered as potential drugs in treatment of LSD, in which accumulation of sphingolipids, especially glycosphingolipids, occurs. Nevertheless, further studies on more advances models are required to test this hypothesis and to assess a therapeutic potential for flavonoids in this group of metabolic brain diseases.

## Introduction

Sphingolipids are ubiquitous components of mammalian cell membranes. Their derivatives, glycosphingolipids (GSLs), constitute a complex group modified by the addition of a carbohydrate unit. GSLs may be divided in two major sets, represented by glucosylceramides (GlcCers) and galactosylceramides (GalCers). Dominant type of glucosylceramides, i.e. gangliosides are the major glycoconjugates found in the nervous system (Schnaar 2005), while galactosylceramides have a much more restricted distribution and are generally confined to myelin and kidney (Schnaar 2009). The essential role of these macromolecules is defined by the fact that defects in genes encoding enzymes of both biosynthesis and degradation of GSLs result in many severe metabolic disorders.

Inborn errors of GSL biosynthesis are at low frequency and result in lethality with a failure of embryos to develop beyond gastrulation (Yamashita et al. 1999), severe epilepsy syndrome (Simpson et al. 2004), or spastic paraplegia (Boukhris et al. 2013). De novo biosynthesis of GSL competes with their formation by salvage pathways using sphingolipid fragments released from the lysosomal compartments. Depending on the cell type, 50 - 90 % of glycosphingolipids are derived from the salvage pathways (Gillard et al. 1998; Tettamanti et al. 2003).

In contrast to sparse GSL biosynthetic diseases, there is a relatively large group found to be an outcome of inappropriate macromolecule catabolism, caused by inherited failure in genes encoding lysosomal proteins. GSLs are degraded along a strictly sequential pathway in humans, and for almost every degradation step, a disease has been described in which the correlated enzyme or activator protein is defective. Lysosomal accumulation of GSLs occurs predominantly in cells and organs that have the highest rates of biosynthesis or uptake of the undegradable sphingolipids and their precursors (Schultz et al. 2011). Such massive accumulation prevents lysosomal function, blocks signaling cascades dependent on Akt-mTOR and Erk (Takamura et al. 2008), and inhibits nutrition delivery through the endolysosomal system, leading to a state of cellular starvation (Jeyakumar et al. 2009).

GLS degradation process occurs in acidophilic cell compartment, endosomal/lysosomal system. Defects in GLS degradation can lead to lysosomal storage disorders (LSDs). Sphingolipidoses are a subgroup of LSDs involving accumulation of sphingolipids and glycosphingolipids (Kolter and Sandhoff 2006). They all occur through two distinct mechanisms of metabolism, defects in biosynthesis or degradation (Platt et al. 2012). One of several factors contributing to the molecular pathogenesis and clinical form of LSDs is the level of residual catabolic activity. Correlation between functional residual catabolic activity and the progression of the lipid storage disorder (Conzelmann and Sandhoff 1983) was basically confirmed for different clinical forms of diseases such as metachromatic leukodystrophy (Leinekugel et al. 1992; Tan et al. 2010), GM2-gangliosidosis (Leinekugel et al. 1992), Gaucher disease (Gieselmann 2005) and Niemann-Pick type A and B diseases (Ferlinz et al. 1995).

Sphingolipid accumulation also occurs in a group of LSDs in which the primary issue is not a lack of a degradation enzyme. Mostly these diseases are associated with problems with trafficking and fusion in the endocytic system, leading to secondary storage of GSLs (Platt et al. 2012). Niemann-Pick type C (NPC), a disease thought to be mainly a disorder of cholesterol transport from the lysosome is an extreme example of this (Rosenbaum and Maxfield 2011).

Among several ways of treatment for LSDs, most effective is enzyme replacement therapy (ERT) (Brady 2006; Lim-Melia and Kronn 2009). Currently, only seven from more than 50 lysosomal disorders are treated with ERT, namely, Gaucher disease, Fabry disease, Pompe disease and mucopolysaccharidosis types I, II, IVA and VI (Lim-Melia and Kronn 2009; Rohrbach and Clarke 2007; Tomatsu et al. 2014). Although such a recombinant enzyme can be delivered to most tissues and organs, it cannot cross the blood–brain-barrier (BBB), and thus, ERT is not effective in treatment of neurological symptoms. Despite the great progress which has been made over the past 20 years, we still lack satisfactory therapies for lysosomal brain diseases.

Until now, only two of LSDs belonging to sphingolipidosis subgroup, Gaucher disease and Fabry disease, can be treated with ERT (Banikazemi et al. 2007; Pastores et al. 2005; Pastores 2010; Tajima et al. 2009). Moreover, an alternative treatment of Gaucher disease is available, which is based on reduction of the substrate synthesis (Hollak et al. 2009). Besides, it was demonstrated that genistein (5, 7-dihydroxy-3- (4-hydroxyphenyl)-4H-1-benzopyran-4-one) can significantly reduce efficiency of synthesis of glycosaminoglycans (GAGs), compounds that accumulate in mucopolysaccharidoses (MPSs) (Piotrowska et al. 2006). The mechanism of action of genistein is based on inhibition of phosphorylation of the epidermal growth factor receptor (EGFR) (Jakobkiewicz-Banecka et al. 2009). As it was shown previously genistein and other flavonoids have also significant impact on expression pattern of genes coding for lysosome metabolism and macromolecule degradation proteins, by the influence of the TFEB regulatory pathway (Moskot et al. 2014; Sardiello et al. 2009). Effects of flavonoids on GAG synthesis and storage, as well as on global gene expression, reported recently (Moskot et al. 2015), revealed that these compounds caused effective reduction in levels of GAG production in fibroblasts, and induced remarkable alterations in profiles of transcripts derived from genes coding for enzymes involved in GAG metabolism and from many other genes. Transcriptomic analyses indicated that, despite certain similarities, there are significant differences between effects of the tested flavonoids (genistein, kaempferol and daidzein used alone, and in combinations) on global gene expression patterns in cells. Moreover, these studies revealed that among the tested compounds, genistein (an isoflavone), kaempferol (a flavonol) and a mixture of these two flavonoids had the most pronounced effects on the regulation of genes’ activities. Interestingly, effects of the mixture of genistein and kaempferol were stronger that those revealed by any of these flavonoids used alone (Moskot et al. 2015). It is worth to mention that these flavonoids can cross the BBB (Tsai 2005), what makes reasonable considering these compounds as potentially useful in the optimization of treatment for neuronopathic forms of LSDs, including sphingolipidoses. Therefore, the aim of this work was to assess effects of flavonoids on expression of genes involved in sphingolipid metabolism.

## Materials and methods

### Cell cultures of fibroblasts

Cell line of Human Dermal Fibroblasts (HDFa) was obtained from the Cascade Biologics, Portland, OR, USA. Fibroblasts were cultured from cryo-preserved cells in Dulbecco’s modified Eagle’s medium (DMEM) containing 10 % fetal bovine serum (FBS) and 1 % antibiotic/antimycotic solution (Sigma-Aldrich, MO, USA) at 37 °C in a humidified atmosphere of 5 % CO_2_. Genistein was synthetized at the Pharmaceutical Research Institute (Warsaw, Poland), while kaempferol and daidzein were obtained from Sigma-Aldrich (Steinheim, Germany). Tested flavonoids were dissolved in dimethyl sulfoxide (DMSO) and added to the indicated final concentrations, as determined in previous studies (Kloska et al. 2011; Piotrowska et al. 2006), to cell cultures. For experimental procedures, cells were plated to a confluence of approximately 80 %. After overnight incubation, culture medium was replaced with fresh medium either flavonoid-free one, containing DMSO at a final concentration of 0.05 % (control, Ctrl), or the one supplemented with appropriate amounts of tested flavonoids. The experimental treatment was carried out for 1, 24 and 48 h period.

### RNA extraction

Total RNA was extracted from cells using the High Pure RNA Isolation Kit (Roche Applied Science, Indianapolis, USA) following the manufacturer’s instructions. The quality and quantity of each RNA sample was evaluated using the RNA 6000 Nano Assay on the Agilent 2100 Bioanalyser (Agilent Technologies Inc., USA).

### Microarray assays for mRNA analysis

Abundance of various mRNAs was measured using Illumina’s Human HT-12 v3 Expression BeadChips (Illumina Inc., CA, USA) [http://www.illumina.com/Documents/products/datasheets/datasheet_humanht_12.pdf]. The BeadChip supports highly efficient whole-genome transcriptional studies targeting more than 25,000 genes with more than 48,000 probes. Probes were designed using the RefSeq and the UniGene databases. Illumina TotalPrep RNA Amplification Kit (Ambion, TX, USA) was used in order to amplify total input RNA. Following linear amplification by T7 RNA polymerase, in vitro synthesized, biotin-labeled cRNA was applied to the BeadChip for hybridization according to the manufacturer’s instructions. BeadChips were scanned using an Illumina BeadArray Reader and the Bead Scan Software (Illumina Inc., CA, USA). The quality of microarray data was controlled by examining raw and adjusted intensity histograms. The detection scores (detection *p* value) were used to determine expressions of each gene using BeadStudio software 2.3.4 (Illumina Inc., CA, USA), which was computed based on *Z*-values of a gene relative to the *Z*-value of negative controls on expression chip. Normalization of gene expression data were done by the GeneSping software 7.3.1 (Agilent Technologies, CA, USA) as the value normalized per chip and per gene with median.

### Data extraction and statistical analysis

Illumina’s Human HT-12v4 Expression BeadChips (Illumina Inc., USA) were used for the microarray analysis of three biological replicates. The assay performance, data extraction and statistical analysis were performed as previously described (Moskot et al. 2014). Gene expression data have been deposited in the NCBI’s Gene Expression Omnibus (GEO, http:www.ncbi.nlm.nih.gov/geo, GEO Series accession number GSE43692 and GSE34074) according to the MIAME standards. The comparison of relative fold change of gene expression levels between flavonoid-treated and non-treated cells was performed. Sphingolipid metabolism pathways were defined according to the KEGG annotation (Kanehisa et al. 2006) (Path: hsa00600, hsa00601, hsa00603 and hsa00604) and AmiGO (GO:0030148, GO:0006665 and GO:0030149).

### Quantitative real-time RT-PCR for mRNA analysis

Quantitative real-time Reverse Transcription PCR (real-time *q*RT-PCR) was performed to measure the mRNA levels of the studied genes using the LightCycler® System 480 (Roche Applied Science, Indianapolis, USA). Total RNA was reverse-transcribed into cDNA using Transcriptor First Strand cDNA Synthesis Kit (Roche Applied Science, IN, USA), according to the manufacturer’s instructions. Quantification of mRNA levels was conducted using LightCycler TaqMan Master (Roche Applied Science, IN, USA). In case of *ARSA*, *ASAH1*, *HEXA*, *NEU1*, *NPC1* and *SUMF1* genes real-time *q*RT-PCR analysis was performed using Real Time ready Custom Panel (cat no. 05532914001, config. no. 90014731, Roche Applied Science, Indianapolis, USA) and LightCyclerH 480 Probes Master (Roche Applied Science, Indianapolis, USA), as determined in previous study (Moskot et al. 2015). However, primers utilized for specific amplification of the *CLN8*, *GBA1*, *GLA*, *GM2A*, *HEXB*, *NPC2*, *PPT1* and *SMPD1* genes were obtained from Thermo Fisher Scientific GmbH. The 2^-∆∆ct^ method was used to determine the relative gene transcript levels after normalization to the reference gene coding for glyceraldehyde-3-phosphate dehydrogenase (*GAPDH*, Ref1), TATA box binding protein (*TBP*, Ref2), phosphoglycerate kinase 1 (*PGK1*, Ref1), and transferrin receptor (*TFRC*, Ref2). Primers and probes of reference genes *GAPDH* (NM_002046), *PGK1* (NM_000291), *TBP* (NM_003194.4) and *TFRC* (ENST00000392396) were bought from Roche Applied Science, IN, USA. Negative controls were tested in parallel. Primers, probes and assay ID are described in Table 1.

**Table 1:** Assay ID, primers and probes used for real-time quantitative Reverse Transcription PCR validation of selected genes

Target	Transcript	Reference gene	Primers	Amplicon		Probe
	Name/Length Sequence ID		Sequence/Length or assay ID	Length (nt)	Region	
GSL metabolism genes	ARSA/1900 ENST00000395624	*PGK1*, *TFRC*	Assay ID: 104759	96	Exons 4-5 932	–
	ASAH1/2618 NM_177924.3	*GAPDH*, *TBP*	F: ACAGTTCTGGAAAATAGCACAAGT/25 R: GGTTGCCTCCCAGGATAAAG/20	98	Exons 9-10 1074	# 57
	CLN8/7185 NM_018941.3	*GAPDH*, *TBP*	F: TCTCCAAGCTGGCCACTATC/20 R: CTGGTTGAGCTTCCAAAACAG/21	127	Exons 2-3 848	# 80
	GBA1/2324 NM_000157.3	*GAPDH*, *TBP*	F: CTTTGTCGACAGTCCCATCA/20 R: CCCTCAGGAATGAACTTGCT/20	102	Exons 9-10 1471	# 9
	GLA/1418 NM_000169.2	*GAPDH*, *TBP*	F: GAAGAGCCAGATTCCTGCAT/20 R: TCCTTCCAGCCTTCTGAGAC/20	77	Exons 1-2 302	# 53
	GM2A/3690 NM_000405.4	*GAPDH*, *TBP*	F: GTGTCCCCCTGAGTTCTCCT/20 R: GTCTGTGCATGGGATCTTGA/20	86	Exons 2-3 384	# 10
	HEXA/2737 NM_000520	*PGK1*, *TFRC*	Assay ID: 117642	63	Exons 5-6 1075	–
	HEXB/2437 NM_000520.4	*GAPDH*, *TBP*	F: CCCCTGGCATTTGAAGGTA/19 R: ACATATTCTCCCCACATACAAGC/23	127	Exons 12-13 1534	# 22
	NEU1/2045 ENST00000375631	*PGK1*, *TFRC*	Assay ID: 126666	102	Exons 2-3 483	–
	NPC1/4741 NM_000271	*PGK1*, *TFRC*	Assay ID: 111681	74	Exons 13-14 2270	–
	NPC2/921 NM_006432.3	*GAPDH*, *TBP*	F: CAGTGAAAAGCGAATATCCCTCTA/24 R: GAGATGAGAAACGATCTGTACTGG/24	111	Exons 3-4-5 472, 549	# 3
	PPT1/2195 NM_001142604.1	*GAPDH*, *TBP*	F: CCAAGGAAACCATTCCCTTAC/21 R: GATGGTCCCCTTCTGTAGCC/20	114	Exons 5-6 720	# 45
	SMPD1/2482 NM_000543.4	*GAPDH*, *TBP*	F: CTATGAAGCGATGGCCAAG/19 R: TGGGGAAAGAGCATAGAACC/20	91	Exons 2-3 1214	# 71
	SUMF1/2159 NM_182760	*PGK1*, *TFRC*	Assay ID: 116675	85	Exons 8-9 1051	–
Other	GAPDH/1338 NM_002046	Reference gene	F: CTCTGCTCCTCCTGTTCGAC/20 R: GCCCAATACGACCAAATCC/19	119	Exons 1-2-3 126	# GAPDH
	PGK1/4887 NM_000291	Reference gene	Assay ID: 102083	78	Exons 4-5 585	# PGK1
	TBP/1851 NM_003194	Reference gene	F: TGAATCTTGGTTGTAAACTTGACC/24 R: CTCATGATTACCGCAGCAAA/20	94	Exons 4-5 824	# TBP
	TFRC/5032 ENST00000392396	Reference gene	Assay ID: 102095	69	Exons 14-15 1820	# TFRC

## Results

### Profiling of expression of genes involved in sphingolipid metabolism with microarrays

**Fig. 1:**
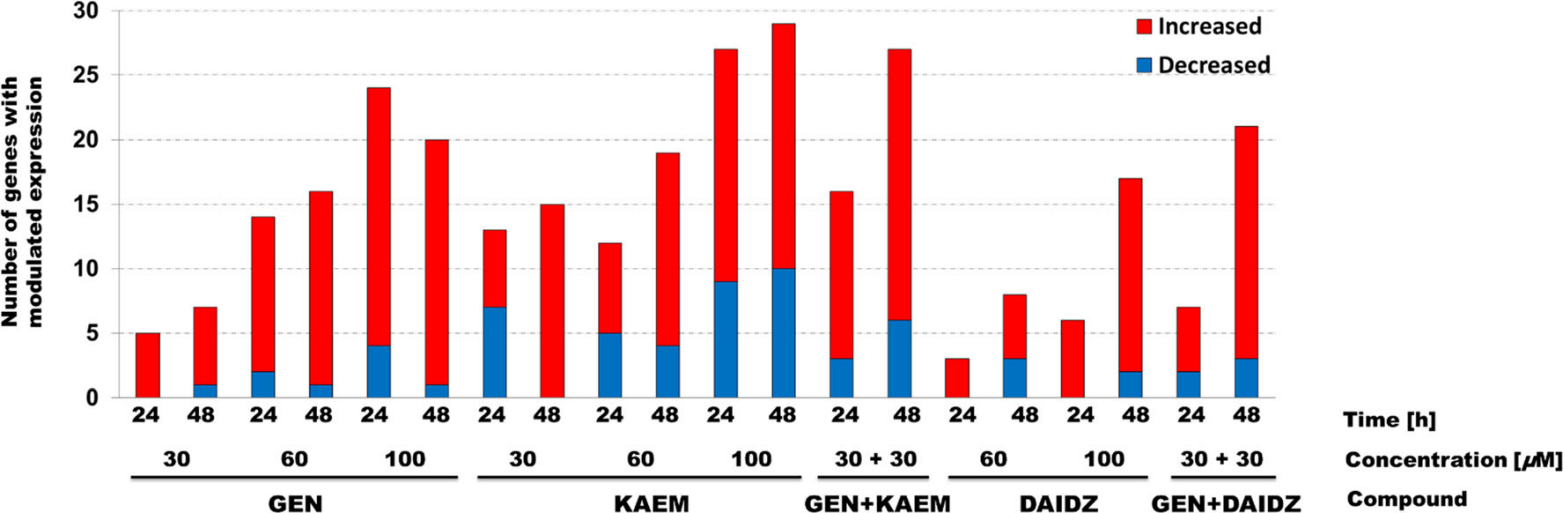
Number of genes revealing altered expression in response to various flavonoids’ treatment type (genistein, kaempferol, daidzein, and mixtures of them as stated), identified in the microarray analysis of GSL metabolism associated transcripts (0.7 ≥ FC ≥ 1.3) of HDFa cells, with the *p*-value <0.05, and *n* ≥ 3

Microarray analysis of transcripts derived from genes coding for sphingolipid metabolism enzymes showed those which expression was identified as different by at least 0.7 and 1.3 fold change (*p* < 0.05) following flavonoids’ exposure in relation to control of non-treated samples of human dermal fibroblasts (HDFa). As discovered in five independent assays, most compound-dependent changes among 121 genes of GSL metabolism occurred at 100 μM kaempferol-treated cells for 48 h (i.e. 29, with 19 up- and 10 down-regulated genes) as shown in Fig. 1. For 24 h treatment with particular flavonoids, 27 genes (i.e. 18 and 9 with a greater than 2-fold increase and decrease in expression, respectively) had modulated expression for 100 μM kaempferol, 20 revealed up-regulation for 100 μM genistein, while 9 were down-regulated for 100 μM kaempferol. After 48 h of exposition to various flavonoids, we found 21 transcripts to be remarkably up-regulated by genistein and kaempferol mix treatment, while ten were found to be down-regulated by 100 μM kaempferol.

Studying carefully the gene expression levels modulated after 24 h of treatment with tested flavonoids, we found one transcript (i.e. *PPAP2A*) in common between all tested conditions, while 5 mRNAs were up-regulated after 48 h of incubation with flavonoids (i.e. *CL4A3BP*, *NEU1*, *PPAP2A*, *SMPDL3A* and *SUMF1*). *PPAP2A* mRNA was therefore included in both 24 and 48 h time course sets. Considering particular flavonoids for both 24 and 48 h treatment, among genes with positively regulated activity, we found 14 for 100 μM genistein (i.e. *ARSA*, *ASAH1*, *COL4A3BP*, *GBA1*, *GBGNT1*, *GM2A*, *HEXA*, *KDSR*, *NEU1*, *NPC1*, *PPAP2A*, *SMPD1*, *SMPDL3A* and *SUMF1*), 13 for 100 μM kaempferol (i.e. *CLN8*, *GLA*, *HEXA*, *KDSR*, *LARGE*, *NEU1*, *NPC1*, *PPAP2A*, *SMPDL3A*, *SPHK1*, *SPHK2*, *ST3GAL1* and *SUMF1*), 5 for genistein and kaempferol mixture (i.e. *GBA1*, *GBGT1*, *PPAP2A*, *SMPDL3A* and *SUMF1*), 3 for 100 μM daidzein (i.e. *ASAH1*, *NEU1* and *PPAP2A*), and 3 for genistein and daidzein mixture (i.e. *GBGT1*, *NEU1* and *PPAP2A*). Taking on account genes with a decreased expression, five transcripts were found to be modulated after both 24 and 48 h of treatment with 100 μM kaempferol (i.e. *B3GNT1*, *ELOVL1*, *SPTLC2*, *ST3GAL6* and *ST6GALNAC5*), while one for genistein and kaempferol mix (i.e. *ST3GAL6*).

When looking at studied conditions, i.e. at least in one experimental state we found several transcripts with increased and/or decreased expression, belonging to various regulation modules with corresponding overlap of some genes between the sets of regulatory processes (Tables 2 and 3). Among those genes we determined several transcripts (i.e. *ARSA*, *ASAH1*, *CLN8*, *GALC*, *GBA1*, *GLA*, *GM2A*, *HEXA*, *HEXB*, *NAGA*, *NEU1*, *NPC1*, *NPC2*, *PPT1*, *SMPD1* and *SUMF1*) being associated with sphingolipids’ disorders (Table 4).

**Table 2:** GSL metabolism genes identified as changed under studied conditions with their corresponding overlap between the sets of regulatory processes (0.7 ≥ FC ≥ 1.3, *n* ≥ 3, with the *p*-value <0.05). Up- and down-regulated genes of HDFa cells after 24 and/or 48 h treatment either with 100 μM genistein, 100 μM kaempferol, genistein-kaempferol of 30 μM each, 100 μM daidzein or genistein-daidzein of 30 μM each

Regulation module	Sphingolipid metabolism pathways										
	GSL biosynthesis - globo series	GSL biosynthesis - lacto and neolacto series	GSL biosynthesis - ganglio series	Lactosylceramide biosynthesis	Glycerolipid metabolism	Ceramide biosynthesis	Sphingosine biosynthesis	Sphingosine degradation	Other sphingolipid metabolism	Intracellular trafficking or fusion	GSL catabolic enzyme modification
Up-regulation	*A4GALT*, *B3GALNT1*, *GBGT1*, *GLA*, *HEXA*, *HEXB*, *NAGA*, *ST3GAL1*	*B3GNT1*, *B3GNT2*, *B3GNT5*, *B4GALT1*, *FUT4*, *FUT6*	*HEXA*, *HEXB*, *SLC33A1*, *ST3GAL1*, *ST3GAL5*	*B4GALT*, *UGCG*	*GLA*, *PPAP2A*, *PPAP2C*	*KDSR*, *LASS2*, *SPTLC1*	*ASAH1*, *KDSR*, *LASS2*	*SPHK1*, *SPHK2*, *SPTLC1*	*ACER3*, *ARSA*, *CLN8*, *COL4A3BP*, *CSNK1G2*, *CYR61*, *GALC*, *GBA*, *GBA2*, *GLA*, *GM2A*, *LARGE*, *NEU1*, *PPAP2A*, *PPAP2C*, *SGMS1*, *SGPP1*, *SMPD1*, *SMPD2*, *SMPDL3A*, *SPHK1*, *SPHK2*, *SPTLC1*, *UGCG*	*NPC1*, *NPC2*	*SUMF1*
Down-regulation	*NAGA*, *ST3GAL2*	*B3GNT2*, *B4GALT4*, *ST3GAL3*, *ST3GAL4*, *ST3GAL6*	*SLC33A1*, *ST3GAL2*, *ST6GALNAC5*	*B4GALT6*, *UGCG*		*SPTLC1*, *SPTLC2*	*SPTLC1*, *SPTLC2*	*SGPL1*, *SPHK1*	*ACER3*, *PRKCD*, *SGMS1*, *SGMS2*, *SGPL1*, *SGPP1*, *SPHK1*, *SPTLC1*, *SPTLC2*, *UGCG*

**Table 3:** Expression patterns of GSL metabolism-associated genes in flavonoids (at least in one experimental condition: 100 μM genistein, 100 μM kaempferol, genistein-kaempferol of 30 μM each, 100 μM daidzein or genistein-daidzein of 30 μM each, for 24 and/or 48 h) treated HDFa fibroblasts analyzed with microarray. Values represent fold change and denote differences for samples treated with tested compounds or their mixture, against untreated samples

Gene symbol	Genistein		Kaempferol		Genistein + Kaempferol		Daidzein		Genistein + Daidzein	
	Time of exposure [h]									
	24	48	24	48	24	48	24	48	24	48
*A4GALT*			1.4 ± 0.1			1.7 ± 0.1		1.5 ± 0.1		1.4 ± 0.0
*ACER3*		1.4 ± 0.4		0.7 ± 0.1						
*ARSA*	1.3 ± 0.2	1.5 ± 0.2		1.3 ± 0.1		1.3 ± 0.1				1.3 ± 0.2
*ASAH1*	1.7 ± 0.8	1.5 ± 0.3				1.9 ± 0.1	1.3 ± 0.0	1.5 ± 0.1		1.8 ± 0.9
*B3GALNT1*						1.4 ± 0.1				
*B3GNT1*			1.6 ± 0.1		1.3 ± 0.1		1.5 ± 0.2			
*B3GNT2*			0.6 ± 0.0	0.5 ± 0.0		1.6 ± 0.2		1.7 ± 0.1		1.9 ± 0.5
*B3GNT5*	1.4 ± 0.0									
*B4GALT1*			1.5 ± 0.2		1.4 ± 0.1					
*B4GALT4*			0.7 ± 0.0							
*B4GALT6*			1.7 ± 0.0			0.7 ± 0.0				
*CLN8*			1.9 ± 0.2	3.3 ± 1.1	1.5 ± 0.3					
*COL4A3BP*	1.5 ± 0.4	1.6 ± 0.4		2.2 ± 0.1		1.8 ± 0.3		1.7 ± 0.1		1.3 ± 0.2
*CSNK1G2*				1.4 ± 0.0						
*CYR61*							1.4 ± 0.3			
*ELOVL1*		0.6 ± 0.1	0.6 ± 0.1	0.7 ± 0.0	0.6 ± 0.1					
*FUT4*						1.4 ± 0.2		1.3 ± 0.1		
*FUT6*	1.4 ± 0.0									
*GALC*						1.7 ± 0.2				1.3 ± 0.2
*GBA1*	1.4 ± 0.4	1.6 ± 0.2			1.4 ± 0.0	1.5 ± 0.2				1.6 ± 0.2
*GBA2*				1.3 ± 0.3						
*GBGT1*	1.9 ± 0.2	1.9 ± 0.3			1.6 ± 0.2	1.9 ± 0.4			1.3 ± 0.3	1.5 ± 0.2
*GLA*			1.5 ± 0.1	1.4 ± 0.0	1.3 ± 0.0					
*GM2A*	1.7 ± 0.4	1.5 ± 0.6								
*HEXA*	1.4 ± 0.2	1.6 ± 0.4	1.4 ± 0.3	1.5 ± 0.3		1.7 ± 0.1				
*HEXB*	1.4 ± 0.1									
*KDSR*	1.8 ± 0.5	1.6 ± 0.6	1.5 ± 0.3	1.5 ± 0.0						
*LARGE*		1.6 ± 0.2	1.3 ± 0.0	1.7 ± 0.2		1.4 ± 0.1				
*LASS2*		1.4 ± 0.2								
*NAGA*				0.6 ± 0.0			1.4 ± 0.5			
*NEU1*	2.9 ± 0.4	2.5 ± 0.3	1.6 ± 0.1	1.5 ± 0.1		2.3 ± 0.1	1.6 ± 0.1	1.6 ± 0.1	1.7 ± 0.2	2.2 ± 0.4
*NPC1*	1.5 ± 0.4	2.3 ± 0.4	1.4 ± 0.4	1.6 ± 0.1	1.4 ± 0.2					
*NPC2*		1.4 ± 0.1				1.4 ± 0.1		1.3 ± 0.1		1.4 ± 0.1
*PPAP2A*	1.4 ± 0.6	1.6 ± 0.5	2.0 ± 0.5	3.6 ± 0.1	2.7 ± 0.2	4.9 ± 0.5	1.5 ± 0.1	1.9 ± 0.1	1.6 ± 0.2	2.0 ± 0.5
*PPAP2C*		1.7 ± 0.0								
*PPT1*	1.4 ± 0.2				1.4 ± 0.1				1.4 ± 0.1	
*PRKCD*	0.7 ± 0.0									
*PRKD1*					1.4 ± 0.1					
*SGMS1*			0.7 ± 0.1			1.3 ± 0.3		1.6 ± 0.1		1.5 ± 0.0
*SGMS2*						0.7 ± 0.0				
*SGPL1*									0.7 ± 0.1	
*SGPP1*			0.6 ± 0.1							1.4 ± 0.4
*SLC33A1*						1.7 ± 0.1		1.3 ± 0.1		1.5 ± 0.3
*SMPD1*	1.4 ± 0.2	1.7 ± 0.2				1.3 ± 0.1		1.5 ± 0.1		1.4 ± 0.1
*SMPD2*	1.6 ± 0.0									
*SMPDL3A*	1.5 ± 0.3	2.3 ± 0.8	1.7 ± 0.1	2.1 ± 0.1	1.5 ± 0.2	2.6 ± 0.2		1.3 ± 0.1		1.6 ± 0.3
*SPHK1*			2.2 ± 0.1	2.0 ± 0.3				0.6 ± 0.0		
*SPHK2*			1.6 ± 0.0	1.4 ± 0.0	1.3 ± 0.1					
*SPTLC1*	1.3 ± 0.2			1.8 ± 0.4						0.6 ± 0.0
*SPTLC2*			0.5 ± 0.1	0.7 ± 0.1	0.7 ± 0.1					
*ST3GAL1*			1.4 ± 0.2	1.5 ± 0.3						
*ST3GAL2*	0.6 ± 0.1			0.5 ± 0.1					0.6 ± 0.1	
*ST3GAL3*				0.6 ± 0.1		0,6 ± 0,0				
*ST3GAL4*	0.7 ± 0.2			0.7 ± 0.1		0.5 ± 0.0		0.6 ± 0.0		0.7 ± 0.1
*ST3GAL5*			1.3 ± 0.1	1.9 ± 0.2		2.7 ± 0.1		1.7 ± 0.2	1.3 ± 0.1	1.7 ± 0.3
*ST3GAL6*	0.7 ± 0.2		0.6 ± 0.2	0.5 ± 0.0	0.7 ± 0.1	0.4 ± 0.1				
*ST6GALNAC5*			0.5 ± 0.0	0.5 ± 0.1		0.5 ± 0.0				0.5 ± 0.1
*SUMF1*	1.6 ± 0.2	1.7 ± 0.3	1.4 ± 0.1	1.6 ± 0.1	1.4 ± 0.1	1.7 ± 0.1		1.5 ± 0.1		1.5 ± 0.2
*UGCG*			0.6 ± 0.2					1.4 ± 0.1

**Table 4:** GSL metabolism genes associated with sphingolipids’ disorders of HDFa cells with modulated expression after 24 and/or 48 h treatment with various flavonoids (100 μM genistein, 100 μM kaempferol, genistein-kaempferol of 30 μM each, 100 μM daidzein and genistein-daidzein of 30 μM each) identified in the microarray analysis (0.7 ≥ FC ≥ 1.3, *n* ≥ 3, with the *p*-value <0.05)

Genes	GSL metabolism disorder	Compounds				
		Genistein	Kaempferol	Genistein + Kaempferol	Daidzein	Genistein + Daidzein
*ARSA*	Metachromatic leukodystrophy (MLD)	√	√	√		√
*ASAH1*	Farber lipogranulomatosis	√		√	√	√
*CLN8*	Neuronal ceroid lipofuscinosis, LINCL variant		√	√		
	Progressive myoclonic epilepsy (PME/EPM)					
*GALC*	Krabbe disease			√		√
*GBA1*	Gaucher disease	√		√		√
	Lewy body dementia (LBD)					
	Progressive myoclonic epilepsy (PME/EPM)					
*GLA*	Fabry disease		√	√		
*GM2A*	Tay-Sachs disease variant AB (GM2 gangliosidosis variant AB)	√				
*HEXA*	Tay-Sachs disease (GM2 gangliosidosis type I)	√	√	√		
*HEXB*	Sandhoff disease (GM2 gangliosidosis type II)	√				
*NAGA*	Schindler/Kanzaki disease				√	
*NEU1*	Sialidosis/Mucolipidosis I	√	√	√	√	√
	Galactosialidosis					
	Progressive myoclonic epilepsy (PME/EPM)					
*NPC1*	Niemann-Pick disease type C1 (NPC1)	√	√	√		
*NPC2*	Niemann-Pick disease type C2 (NPC2)	√		√	√	√
*PPT1*	Neuronal ceroid lipofuscinosis (CLN1, INCL)					√
	Progressive myoclonic epilepsy (PME/EPM)					
*SMPD1*	Niemann-Pick disease (NPD) type A and B	√		√	√	√
*SUMF1*	Multiple sulfatase deficiency (MSD)	√	√	√	√	√

### Real-time qRT-PCR analysis of expression of genes involved in sphingolipid metabolism

**Fig. 2:**
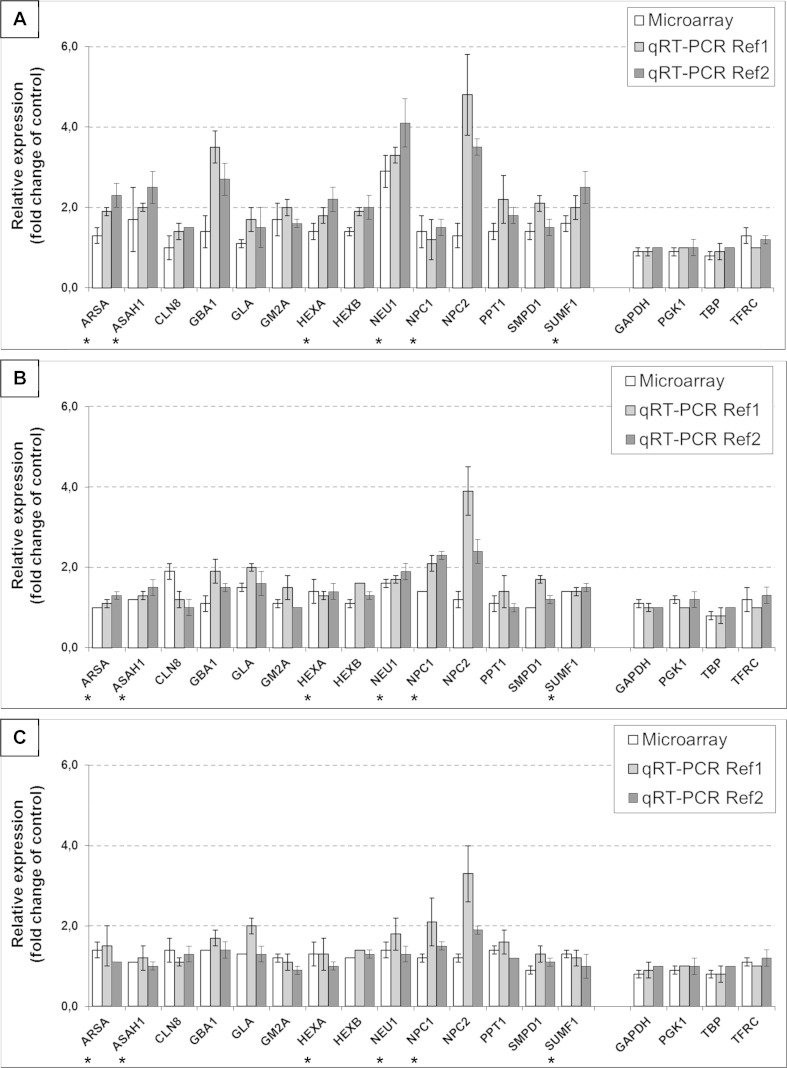
Effect of genistein (100 μM, **a**), kaempferol (100 μM, **b**), and mix of genistein and kaempferol (30 μM each, **c**) on mRNA level of selected genes up-regulated with an expression ratio greater than 1.3-fold, and with statistical significance set at *p* ≤ 0.05, involved in sphingolipid metabolism in HDFa cells after 24 h of treatment. Two types of analyses were made: microarray gene expression studies (*white columns*) and real-time *q*RT-PCRs (*light* and *dark grey columns*), where the data represent averaged values ±SD from *n* ≥ 3 and denote significant differences for samples treated with tested compound/s against non-treated samples, with respect to reference genes of constant expression level: *GAPDH* (Ref 1) and *TBP* (Ref 2) for *CLN8*, *GBA1*, *GLA*, *GM2A*, *HEXB*, *NPC2*, *PPT1* and *SMPD1*, while *PGK1* (Ref 1) and *TFRC* (Ref 2) for *ARSA*, *ASAH1*, *HEXA*, *NEU1*, *NPC1*, and *SUMF1*, respectively. *Results based on raw data from Moskot et al. (2015)

Real-time quantitative Reverse Transcription PCR (real-time *q*RT-PCR) was used to confirm a set of gene expression changes observed in the microarray analysis. To choose the most stable genes as internal references for real-time *q*RT-PCR data normalization, two candidates - *GAPDH* (Ref1) and *TBP* (Ref2) for *CLN8*, *GBA1*, *GLA*, *GM2A*, *HEXB*, *NPC2*, *PPT1* and *SMPD1*, while other two - *PGK1* (Ref1) and *TFRC* (Ref2) for *ARSA*, *ASAH1*, *HEXA*, *NEU1*, *NPC1*, and *SUMF1* genes, respectively, were selected according to their expression levels detected in the microarray studies. From 59 GSL metabolism genes regulated under tested conditions (Table 3), 14 from 16 genes coding for enzymes involved in glycosphingolipid metabolism disorders (as shown in Table 4) were experimentally validated (Fig. 2). For the two other genes, *GALC* and *NAGA* designing of intron spanning primers was not possible, therefore they were not included in the analysis. Basically, the real-time *q*RT-PCR and microarray methods showed a good agreement on regulated genes (Fig. 2).

## Discussion

Since molecular mechanisms of LSDs are rather well understood (relative to many other diseases of this nature), and as they were among the first genetic diseases for which effective - at least to some extent - treatment became possible, these disorders may be considered as models in inherited metabolic diseases. There are several LSDs, in which accumulation and storage of sphingolipids occur as the primary cause of symptoms (Sandhoff 2013).

In the light of problems with crossing BBB, it appears crucial to look for potential drugs that could be used in pathological sphingolipid storage. One group of such potential drugs are flavonoids, such as genistein (an isoflavone), kaempferol (a flavonol) and daidzein (an isoflavone), compounds which were reported previously to partially inhibit GAG synthesis and to reduce GAG storage in fibroblasts derived from patients suffering from mucopolysaccharidoses, metabolic disorders belonging to LSDs (Arfi et al. 2010; Kloska et al. 2011). Since potentially many genes involved in synthesis of various biologically active molecules could be controlled by the EGF-dependent pathways, and at the same time some other transcripts regulating lysosomal administration are regulated by the TFEB pathway, we asked if previously selected and studied flavonoids can also influence expression of genes coding for enzymes required for sphingolipid metabolism.

In this report, we demonstrate that flavonoids influence expression of dozens of genes involved in sphingolipid metabolism. In general, the DNA microarray analysis indicated that kaempferol, genistein and mix of these two compounds profiled the expression of the greatest number of genes (Fig. 1). When looking at particular studied conditions, we found numerous of transcripts with altered expression belonging to various regulation modules, such as: GSL biosynthesis - globo series, GSL biosynthesis - lacto and neolacto series, GSL biosynthesis - ganglio series, lactosylceramide biosynthesis, glycerolipid metabolism, ceramide biosynthesis, sphingosine biosynthesis, sphingosine degradation, other sphingolipid metabolism genes, intracellular trafficking or fusion GSL catabolic enzyme modification, with corresponding overlap of genes between these sets of regulatory processes (Table 2). Additionally, we conclude that 59 among 121 genes of GSL metabolism were regulated at least in one experimental condition (Table 3). What is interesting, 16 genes from them are associated with well-known sphingolipids disorders (Table 4).

Previous studies indicated that flavonoids, particularly genistein and kaempferol, modified expression of genes coding for metabolism of GAGs in a specific manner. Namely, expression of many (but not all) genes which products are involved in synthesis of GAGs were down-regulated while a few were up-regulated, whereas vast majority of transcripts derived from genes coding for GAG-degradation enzymes were more abundant after treatment with genistein, kaempferol or their mixture (Moskot et al. 2014). In the case of GSL metabolism, the picture is more complicated. In both groups, up- and down-regulated by flavonoids, there are genes coding for enzymes involved in synthesis and degradation of particular lipids’ derivatives. Moreover, in each particular pathway for production and degradation of specific class of GSLs, there are always stimulated and inhibited genes coding for enzymes operating in both opposite pathways. One might predict that inhibition of a crucial step in synthesis of particular GSL may results in impairment of the whole process, even if other steps are stimulates, according to the bottleneck mechanisms, i.e. the restriction of the efficiency of the whole pathway by the slowest reaction. However, such a hypothesis requires experimental verification.

It does not escape our attention that fibroblasts are not the best models for genetic and biochemical changes in neurons, while diseases caused by GSLs’ accumulation cause brain dysfunctions. However, for in vitro experiments with the microarray technology, which allowed us to monitor expression of many genes at the same time but which also require relatively large amount of biochemically good quality material isolated from actively growing cells, human fibroblasts appeared to be the optimal choice. Other possible experimental systems would have severe restrictions in the availability of the material and viability of cells. Nevertheless, it is obvious that any further studies which might lead to determination if flavonoids can be considered as potential drugs for management of GSL-storage diseases should involve either cultures of cells types which are models more closely resembling physiology of the brain or animal models for particular diseases.

In summary, our results indicate that expression of several genes involved in sphingolipid synthesis can be regulated by tested flavonoids. Therefore, it is tempting to speculate that they may be considered as potential drugs in treatment of LSD, in which accumulation of sphingolipids, especially glycosphingolipids, occurs. Nevertheless, further studies on more advances models are required to test this hypothesis and to assess a therapeutic potential of flavonoids in this group of metabolic brain diseases.
